# X-ray electron density analysis of chemical bonding in permanent magnet Nd_2_Fe_14_B

**DOI:** 10.1107/S2052252525007602

**Published:** 2025-09-29

**Authors:** Emilie Skytte Vosegaard, Jacob Svane, Bo Brummerstedt Iversen

**Affiliations:** a Center for Integrated Materials Research, Department of Chemistry and iNANO Aarhus University Langelandsgade 140 8000Aarhus Denmark; ESRF, France

**Keywords:** X-ray electron density, magnetism, synchrotron single-crystal X-ray diffraction, inorganic materials

## Abstract

Chemical bonding determines the intrinsic properties of materials, but despite the super-strong metallic magnet Nd_2_Fe_14_B being a vital compound in modern life, the local chemical environment is not well understood. Nd_2_Fe_14_B has a very complex crystal structure with six independent Fe sites, two independent Nd sites and one B site, which in concert are responsible for the extremely high magnetic moment. Dense inorganic materials with excellent crystal quality and heavy atoms represent a strong challenge to X-ray charge density analysis, and indeed Nd_2_Fe_14_B has a mere suitability factor of 0.02 compared with 3–5 for typical organic molecular crystals. Here, we report high-energy (λ = 0.2482 Å) 25 K single-crystal synchrotron X-ray diffraction data suitable for multipole modelling of the X-ray charge density. The X-ray electron density shows local anisotropy in the bonding environment of the Fe atoms, and topological analysis quantifies that the Nd atoms are positive (∼+1), one Fe atom and B are negative (−1.7 and −0.44, respectively), and the remaining Fe atoms are close to neutral (±0.1). The *d* orbitals of all Fe atoms are close to being evenly populated, and bonding analysis establishes a multidirectional ‘metal-like’ framework. It is found that a single Fe atom is crucial for the 3D framework of the magnetic structure. Through structural refinement of synchrotron single-crystal X-ray diffraction data at 25 K, 100 K, 200 K and 300 K, anisotropic displacement parameters are obtained, and the Debye temperature is estimated to be 345–383 K.

## Abstract 2

Short-wavelength high-resolution synchrotron single-crystal X-ray diffraction data measured at 25 K are used to model the electron density of the super-strong magnet Nd_2_Fe_14_B and quantify its complex chemical interactions.

## 1. Introduction

Magnets are omnipresent in modern society from engines to electronics and much in between. The discovery of the super-strong magnet Nd_2_Fe_14_B in 1982 revolutionized magnetic research (Croat *et al.*, 1984 ▸; Sagawa *et al.*, 1984 ▸), and still today it has the highest known energy product. It is produced on a large scale and applications span from windmills to electrical cars – it is an essential material for the green transition (Hioki, 2021 ▸; Wu *et al.*, 2018 ▸). Following the discovery, the crystal structure was established by single-crystal X-ray diffraction (SCXRD) in 1984 (Shoemaker *et al.*; 1984 ▸), but since then research has largely focused on technological applications and less on understanding the complex atomic structure fundamentally responsible for the outstanding properties. It is striking that no studies so far have concerned the basic electronic structure of the material. It is the nature of the chemical bonding between atoms that induces the specific properties of a material, but for Nd_2_Fe_14_B there is an absence of the chemical bonding analysis which otherwise often drives modern materials development. The reasons for this knowledge gap are probably many, but one is the complexity of the crystal structure where the unit cell contains 68 open-shell atoms making accurate theoretical modelling extremely challenging due to numerous closely spaced energy states. At this point there is limited insight into the fundamental chemical interactions and atomic properties of Nd_2_Fe_14_B.

The electronic structure of a crystalline material can be obtained theoretically, *e.g.* through periodic density functional theory calculations (Gu & Ching, 1987 ▸), and experimentally the crystal electron density can be determined from modelling of very accurate high-resolution X-ray diffraction data (Stokkebro Schmøkel, Overgaard & Brummerstedt Iversen, 2013 ▸). For X-ray diffraction the dense heavy atom nature leads to substantial absorption and extinction effects in the data, but such challenges may be overcome using high-energy synchrotron radiation and small crystal specimens coupled with very low temperature measuring conditions (Tolborg & Iversen, 2019 ▸). Helium cooling allows data to be recorded to very high resolution due to limited thermal damping of the high-order intensity. High-order data are vital for separating valence electron effects from thermal motion effects (Iversen *et al.*, 1999 ▸). However, the more fundamental challenge is that the scattering signal from the relatively few valence electrons is very weak compared with the signal from the electron-rich atomic cores. This puts strong demands on the accuracy of the data if the chemical bonding (valence electrons) is to be studied. The suitability factor quantifies the ratio between valence electrons and core electrons, and for typical organic molecular crystals it has a value of 3–5 (Stevens & Coppens, 1976 ▸). For extended inorganic solids previously studied by X-ray electron density analysis such as TiS_2_ (Kasai *et al.*, 2018 ▸), CoSb_3_ (Stokkebro Schmøkel, Bjerg *et al.*, 2013 ▸), FeSb_2_ (Grønbech *et al.*, 2020 ▸) and ZnSb (Grønbech *et al.*, 2024 ▸) it is 0.01–0.1, whereas for Nd_2_Fe_14_B the suitability has a value of 0.02. The crystal complexity and heavy-atom nature of Nd_2_Fe_14_B possibly makes this the most difficult X-ray electron density study so far. To succeed we first embarked on synthesis of very high quality single crystals using the Czochralski technique, and then collected X-ray diffraction data at 25 K using high-energy X-rays from the SPring8 synchrotron in Japan.

## 2. Experimental

### 2.1. Synthesis

For growth, a 17.4 g sample composed of 51 at% Nd, 44 at% Fe and 5 at% B (Susner *et al.*, 2017 ▸) was loaded into a pyrolytic BN crucible which was placed in a graphite susceptor. Before loading the BN crucible, the oxide layer which had formed on top of the Nd was scratched off. The graphite susceptor was placed in a copper coil with five windings which was placed in an ADL-type induction furnace. The furnace was evacuated to ∼2 × 10^−2^ mbar and filled with He to ambient pressure five times before being pressurized to 200 psi with He. The samples were heated to ∼1200°C and left to homogenize for 30 min after melting to ensure an even distribution of all elements throughout the melt. The crucible was rotated at 1.5 rpm to give a better heat distribution throughout the melt. After homogenization a tungsten wire was lowered into the melt and a Czochralski pull was begun with a pulling rate of ∼8 mm h^−1^. The growth was carried out and a rod with a length of ∼3 cm was obtained. During the growth, some impurity was observed to be present on top of the melt. This impurity is believed to originate from some oxidation of Nd before the inert atmosphere was established; the impurities were not believed to have affected the growth as rotation of the crucible ensured that the impurities were pushed towards the crucible edge. The rod was removed from the furnace and submerged in oil in order to avoid oxidation of the grown crystal. Submersion in oil was chosen as some light coloration was observed on the outside of the crystal. It is unknown whether this coloration arose from some of the impurities observed on the melt during growth or from oxidation of the rod after exposure to ambient air. To obtain a crystal for SCXRD measurements, the rod was snapped in two pieces. A sharp razor was used to apply pressure to the newly exposed surface to scrape off crystallites. The crystallites were transferred to a glass slide and covered in oil to avoid oxidation.

### 2.2. SCXRD measurements

Data were measured on a 25 µm × 60 µm × 130 µm crystal at SPring-8 at the BL02B1 beamline using a wavelength of 0.2482 Å. Datasets were collected at 300 K, 200 K, 100 K and 25 K using liquid N_2_/He cryostats to control the temperature. The beamline is equipped with a quarter χ goniometer and a Pilatus3 X 1M CdTe detector (Krause *et al.*, 2020 ▸). For the 300 K, 200 K and 100 K data, three 180° ω scans were collected at χ values of 0°, 25° and 45° for a 2θ value of 0°. For the 300 K dataset an exposure time of 0.5 s/frame was used while for the 200 K and 100 K an exposure time of 0.25 s was used. A scan width of 0.1°/frame was used resulting in a total of 5400 frames collected for each of the three temperatures. A low-angle 25 K dataset was collected with the same strategy as the 200 K and 100 K datasets, with an additional full 180° ω scan in the χ = 0° setting (1800 extra frames) and the first 90° of the ω scan for χ = 25° (900 frames). To obtain a higher resolution a high-angle dataset was collected at 25 K with χ values of 0°, 25° and 45° and a 2θ value of 20°, resulting in a total of 13500 frames collected at 25 K. Data obtained from SPring8 were converted to Bruker format using the Pilatus3 frame conversion software developed by Krause (https://github.com/LennardKrause/). Data reduction was performed in the *APEX5* GUI for the program *SAINT* (Bruker, 2019 ▸) with the recurrence background setting. *SADABS* (Krause *et al.*, 2015 ▸) was used for scaling and absorption correction (μ = 4.3, μ*r* = 0.15) of the integrated data. The isotropic absorption correction factor, μ*r*, is calculated from the tabulated linear absorption coefficient, μ = 4.3 mm^−1^ [calculated as an average of the FPRIME and BRENNAN values from *WinGX* (Farrugia, 2012 ▸)] and a projected isotropic radius (*r* = 0.035 mm^−1^) estimated by equating the crystal block volume to the volume of a sphere. Two datasets were obtained for low- and high-angle data at 25 K. The *SORTAV* (Blessing, 1997 ▸) program was used for merging the two datasets, as well as rejection of outliers, merging of the equivalent reflections and to obtain estimates of their uncertainties. Reflections with *d*-spacing higher than *d*_max_ = 4 Å (sin θ/λ_min_ = 0.125 Å^−1^) were rejected, due to only partial detection, as they were hidden by the beam stop shadow or hit spaces between detector panels [in total 6 reflections: (101), (110), (002), (111), (200) and (112)]. An initial estimate of the resolution limit was *d*_min_ = 0.27 Å (sin θ/λ_max_ = 1.85 Å^−1^) since significant reflections were available to this limit, but the resolution was later reduced to *d*_min_ = 0.33 Å (sin θ/λ_max_ = 1.5 Å^−1^) for better reflection statistics. For the datasets at 100 K, 200 K and 300 K all reflections until a resolution of *d*_min_ = 0.4 Å (sin θ/λ_max_ = 1.25 Å^−1^) were used.

### 2.3. Structure solution and IAM refinement

The structure was solved and refined using *SHELXT* (Sheldrick, 2015*a* ▸) and *SHELXL* (Sheldrick, 2008 ▸; Sheldrick, 2015*b* ▸) in *Olex2* (Dolomanov *et al.*, 2009 ▸). The independent atom model (IAM) structure included free refinement of atomic coordinates and anisotropic atomic displacement parameters (ADPs), up to nine parameters per atom (*x*, *y*, *z* and six *U*_*ij*_ ADPs) when allowed by the site symmetry. All occupancies refined to 1 within ±0.001 and were fixed to 1.0. Crystallographic information is given in Table 1 ▸.

### 2.4. Multipole modelling

For electron density analysis a multipole model (MM) was refined in *XD2016* (Volkov *et al.*, 2016 ▸) against the merged 25 K dataset using the Volkov & Macchi (unpublished work) databank for radial functions. Only the valence electrons were allowed to deform in the multipoles, so the following choice of valence was made: the outermost *s* and *f* electrons (6*s*^2^4*f*^4^) were considered valence for Nd, for Fe the two 4*s* electrons were given a separate monopole and κ, and the six 3*d* electrons were allowed to deform, while the 2*s* and 2*p* electrons were considered valence for B. The lanthanides are usually found in their +III oxidation state, but since no obvious charge count for the remaining ions would balance this, all atoms were kept neutral in the initial model. All symmetry-allowed multipoles up to the hexadecapole level (*l*_max_ = 4) were allowed to refine along with atomic coordinates and anisotropic ADPs. A test for anharmonic motion on Nd and Fe was performed, but due to low significance, high correlation with harmonic vibrational parameters (>80%) and no improvement in quality parameters it was deemed irrelevant for the model. For a better description of Nd, a separate κ and a dipole were refined for the outermost orbitals of the Nd core, specifically the diffuse 4*d*^10^ and 5*s*^2^5*p*^6^ (see the supporting information). In the final model, the κ parameters were fixed and the scale factor was manually optimized (reduced by ∼0.2%) (Meindl & Henn, 2008 ▸), accounting for an insufficient correction for absorption and/or anomalous dispersion correction of the Nd atoms (Meurer *et al.*, 2024 ▸). Attempts to model the 4*f* electrons on Nd explicitly using multipoles up to the *l*_max_ = 6 level in *Jana2020* (Petříček *et al.*, 2023 ▸) gave no improvements to the model, so the *XD2016l*_max_ = 4 model was used for further analysis (see discussion in the supporting information).

**Figure 1:**
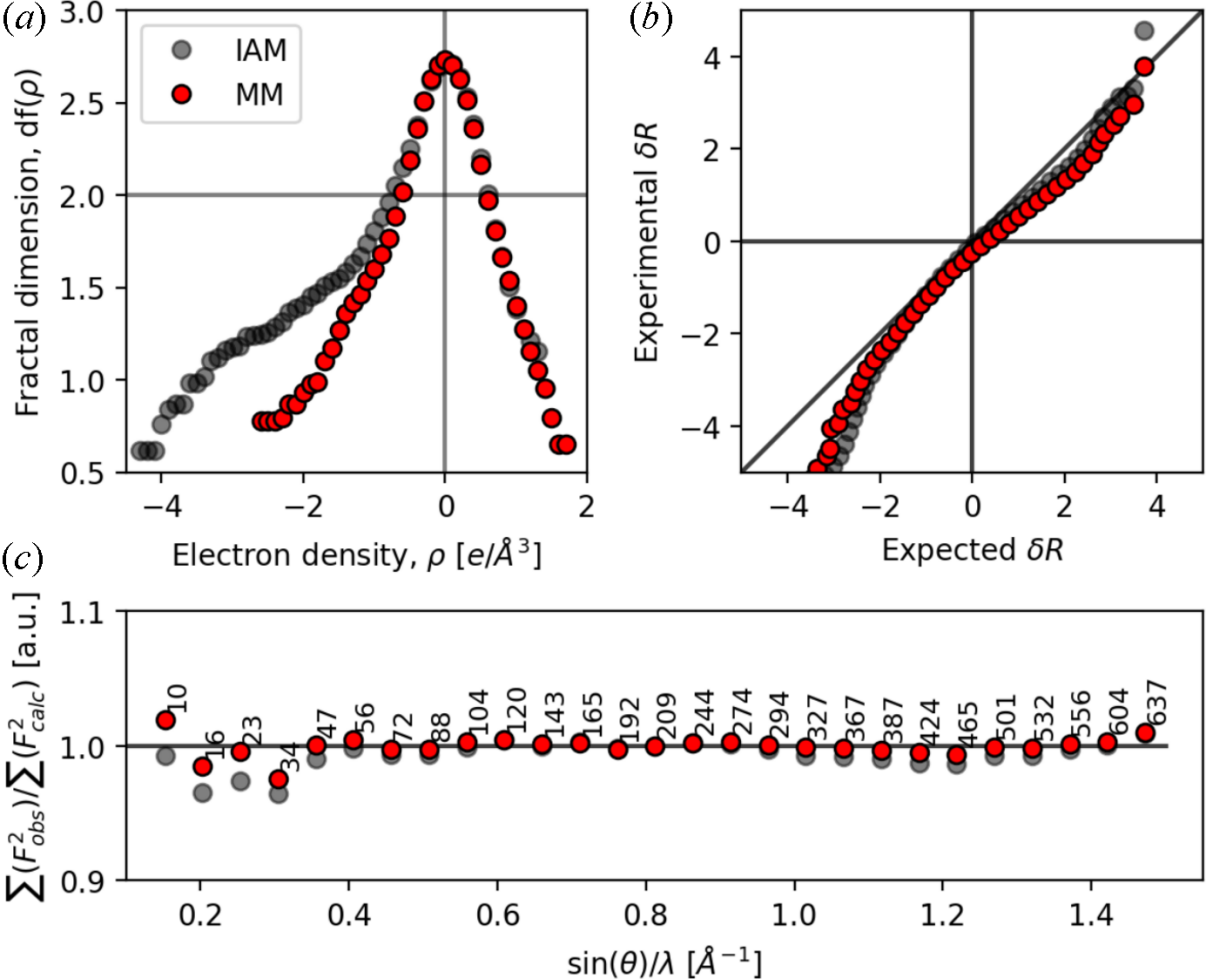
(*a*) Fractal dimension plot, (*b*) normal probability plot [δ*R* = (*F*^2^_obs_ − *F*^2^_calc_)/σ(*F*^2^)], and (*c*) *F*^2^_obs_/*F*^2^_calc_ plot for the MM (red) and IAM (grey) of Nd_2_Fe_14_B.

The κ values were 1.049/1.026 for the Nd valence/core, 1.046/1.006 for Fe 3*d*/4*s* valence and 0.990 for B. The largest correlation coefficient between parameters was 0.80 between the core and valence dipole on Nd. The highest peaks (1.25 e Å^−3^ and 1.08 e Å^−3^) and deepest holes (−2.13 e Å^−3^ and −1.17 e Å^−3^) in the residual density were found at a distance less than 0.5 Å from Nd1 and Nd2, respectively, suggesting an incomplete description of the density in the vicinity of the Nd atoms. The largest residuals on Fe atoms are 0.70 e Å^−3^ 0.5 Å from Fe6 and −1.04 e Å^−3^ 0.5 Å from Fe4. Fig. 1 ▸(*a*) shows a systematic narrowing of the fractal dimension plot when comparing the IAM and MM. The normal probability plot in Fig. 1 ▸(*b*) shows no systematic changes, while the *F*^2^_obs_/*F*^2^_calc_ plot in Fig. 1 ▸(c) shows improvements for the low-angle reflections (sin θ/λ < 0.4 Å^−1^), as expected for a better description of the valence deformation in the MM.

**Figure 2:**
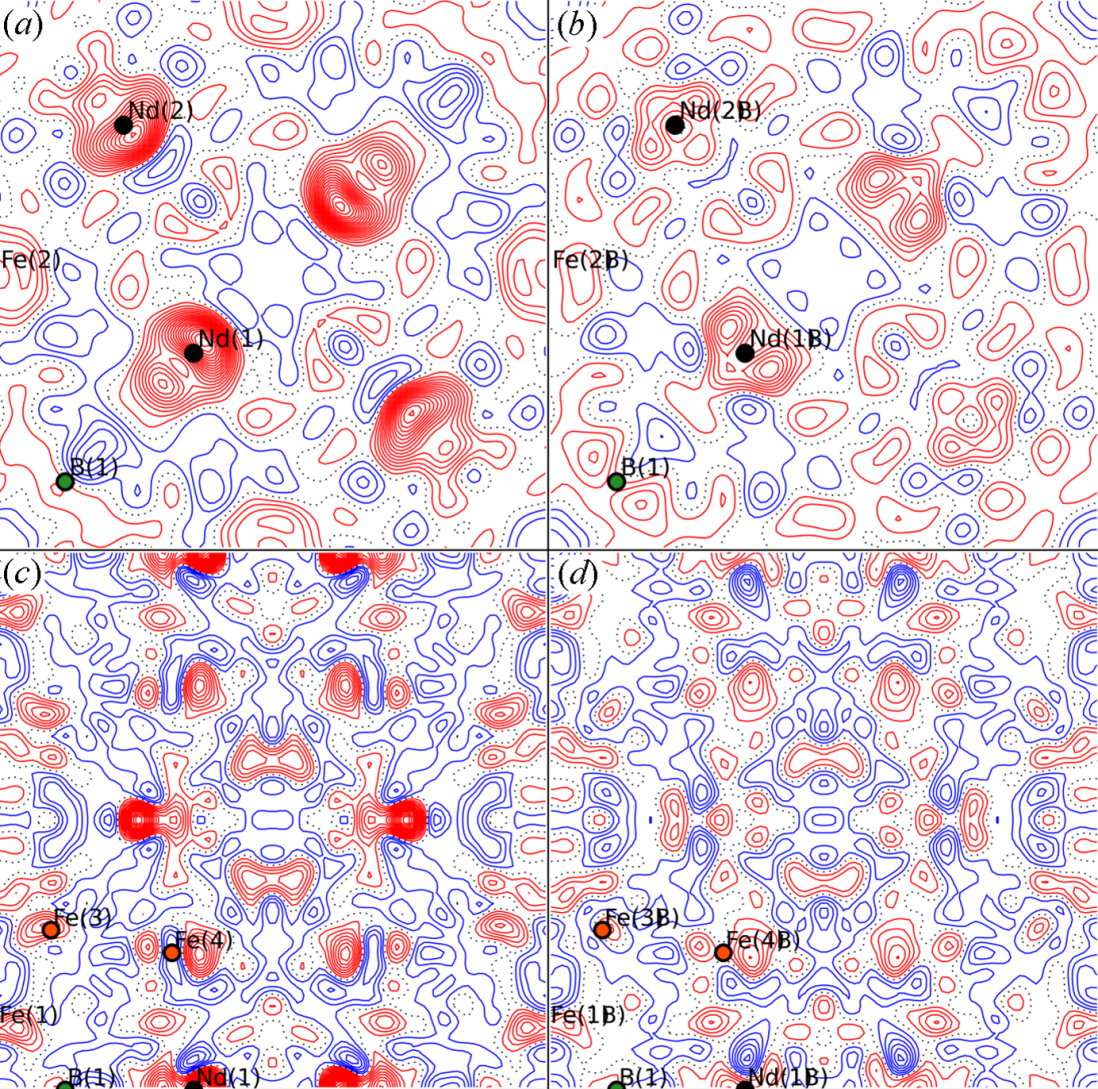
Residual density maps of Nd_2_Fe_14_B in the (*a*)–(*b*) (001) and (*c*)–(*d*) (110) planes of the unit cell. (*a*) and (*c*) show the IAM, and (*b*) and (*d*) show the MM. The resolution is truncated at 0.7 Å^−1^ to emphasize valence scattering. Positive (blue), negative (red) and zero (black) contours are shown at the 0.1 e Å^−3^ level.

From the residual density maps of the IAM [Figs. 2 ▸(*a*) and 2 ▸(*c*)] and MM [Figs. 2 ▸(*b*) and 2 ▸(*d*)] in the (001) and (110) planes in Fig. 2 ▸, it is apparent that the residuals near the atomic sites significantly improve in the MM. The residual density provides the discrepancy between the observed and modelled density, and the improvement is seen by a pronounced reduction in the extent, as well as height and depth of the residuals (number of contour lines) in the vicinity of atomic centres. For example, a deep hole is placed near Nd1, seen as an excessive number of negative (red) contours in the IAM [Fig. 2 ▸(*a*)], a feature which is much shallower in the MM [Fig. 2 ▸(*b*)]. Similarly, a positive (blue) feature near Nd2 in the IAM [three contours in Fig. 2 ▸(*a*)] is almost eliminated in the MM [one contour in Fig. 2 ▸(*b*)], while also the extent and depth of the negative (red) contours are significantly reduced. The same is seen for the Fe atoms in Figs. 2 ▸(*c*)–2 ▸(*d*), where *e.g.* Fe3 shows a shift from five red contours in the IAM to only two red contour lines in the MM, and the positive–negative–positive feature at Fe4 in the IAM is eliminated to only exhibit reduced negative residuals in the MM. Importantly, the MM allows for extraction of the electron density for analysis.

## 3. Results and discussion

### 3.1. Structure and ADPs

Nd_2_Fe_14_B (*M* = 1081 g mol^−1^) crystallizes in the tetragonal space group *P*4_2_/*mnm*, with unit-cell parameters *a* = 8.8143 (2) Å and *c* = 12.2117 (2) Å at 300 K, and four formula units in the unit cell. Note that a spin-reorientation phase transition happens at 135 K resulting in a monoclinic magnetic space group *Cm* to account for a 37° tilted magnetic moment. Here we report the structure at four temperatures (25 K, 100 K, 200 K and 300 K) of a non-magnetized sample below the Curie temperature of 585 K (Sagawa *et al.*, 1984 ▸). In this 275 K temperature range the unit cell shows linear, though minute, changes of less than 1% increase in volume, and linear expansion coefficients of 0.8 (5) × 10^−6^ K^−1^ and 6.6 (7) × 10^−6^ K^−1^ along the *a* and *c* directions, respectively. This is consistent with Herbst *et al.* (1985 ▸) observing significant unit-cell changes only above the Curie temperature.

The asymmetric unit contains nine atoms: Nd1 (Wyckoff site: 4*f*), Nd2 (4*g*), Fe1 (4*e*), Fe2 (4*c*), Fe3 (8*j*), Fe4 (8*j*), Fe5 (16*k*), Fe6 (16*k*) and B1 (4*f*). The reported unit cell and atomic positions (Table 2 ▸) are consistent with Herbst *et al.* (1984 ▸) (*a* = 8.80 Å, *c* = 12.19 Å) and Shoemaker *et al.* (1984 ▸) [*a* = 8.804 (5) Å and *c* = 12.205 (5) Å]. Atomic positions are in excellent agreement with Shoemaker *et al.* (1984 ▸) for Nd and Fe differing only on the fourth decimal, while B coordinates differ by 0.002.

**Figure 3:**
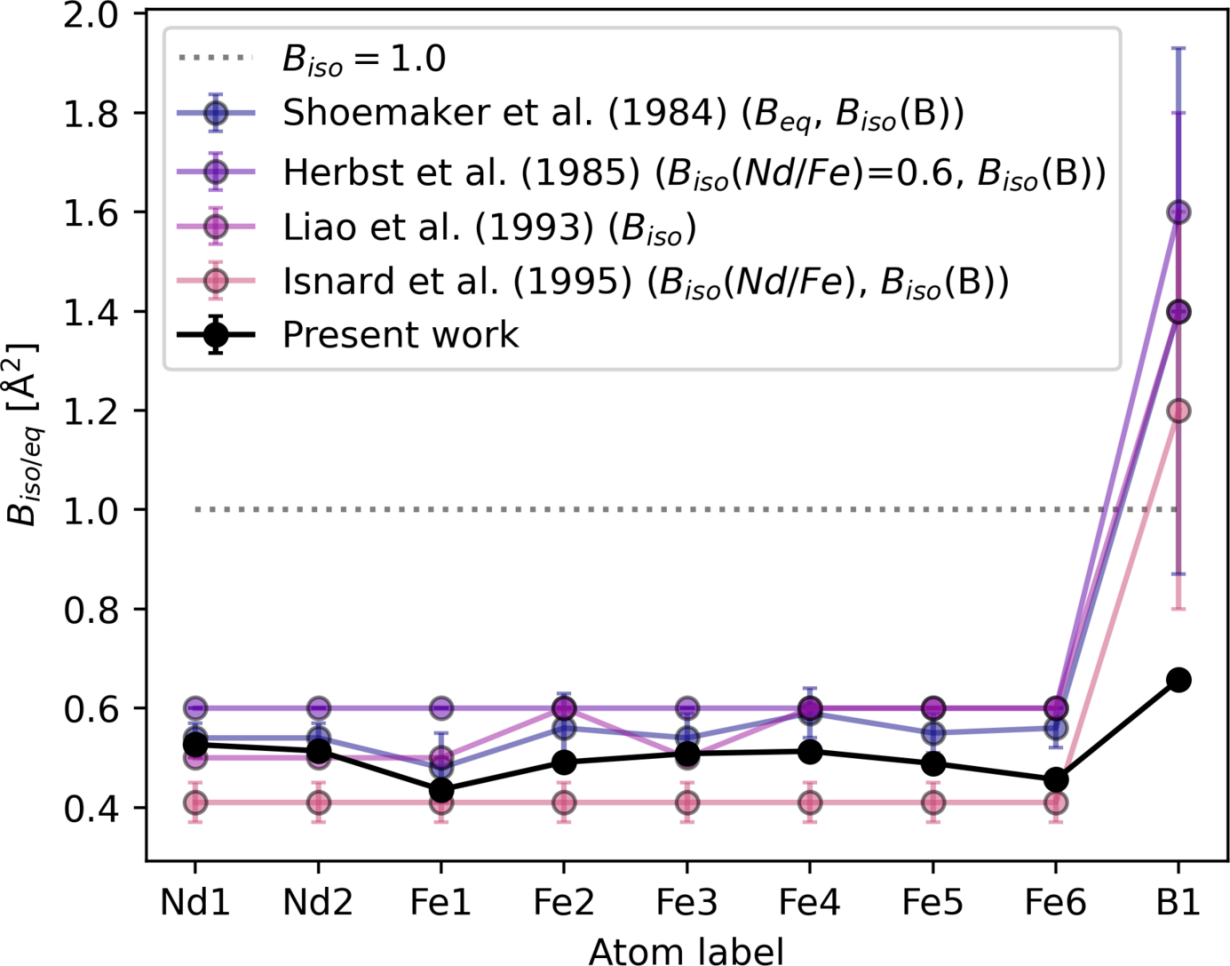
Isotropic or equivalent vibrational parameters (*B*_iso/eq_) as reported by Liao *et al.* (1993 ▸) (ICSD code 41393), Herbst *et al.* (1985 ▸) (ICSD code 44294), Shoemaker *et al.* (1984 ▸) (ICSD code 48143), Isnard *et al.* (1995 ▸) (ICSD code 80971) and the 300 K structure in this study. The dotted grey line shows the commonly used *B*_iso_ = 1.0 line.

Of the 31 entries for Nd_2_Fe_14_B with the *P*4_2_/*mnm* space group at 300 K in the ICSD, only four report isotropic (*B*_iso_) or anisotropic (*B_ij_* or *B*_eq_) vibrational parameters that are different to *B*_iso_ = 1. *B*_eq_’s for the structure presented here can be seen in Table 2 ▸, and Fig. 3 ▸ shows a comparison of the reported ADPs for Nd_2_Fe_14_B in the ICSD. Shoemaker *et al.* (1984 ▸) refined separate *B_ij_*’s for all Nd and Fe, and *B*_iso_ for B, with the same relative behaviour as the present study, while slightly overestimating the vibrations (on average 10% for Nd and Fe, and >100% for B). Herbst *et al.* (1985 ▸) refined one common *B*_iso_ for Nd and Fe, and one for B, overestimating the vibrations for all atoms, while Isnard *et al.* (1995 ▸) underestimated the vibrations of Nd and Fe by refining one *B*_iso_ for each type of atom, and fixed *B*_iso_ = 1.2 Å^2^ for B. Liao *et al.* (1993 ▸) refined *B*_iso_’s for all atoms, but gave no uncertainties for the parameters. In general, the B vibrations are heavily overestimated by a factor of 2–3.

**Figure 4:**
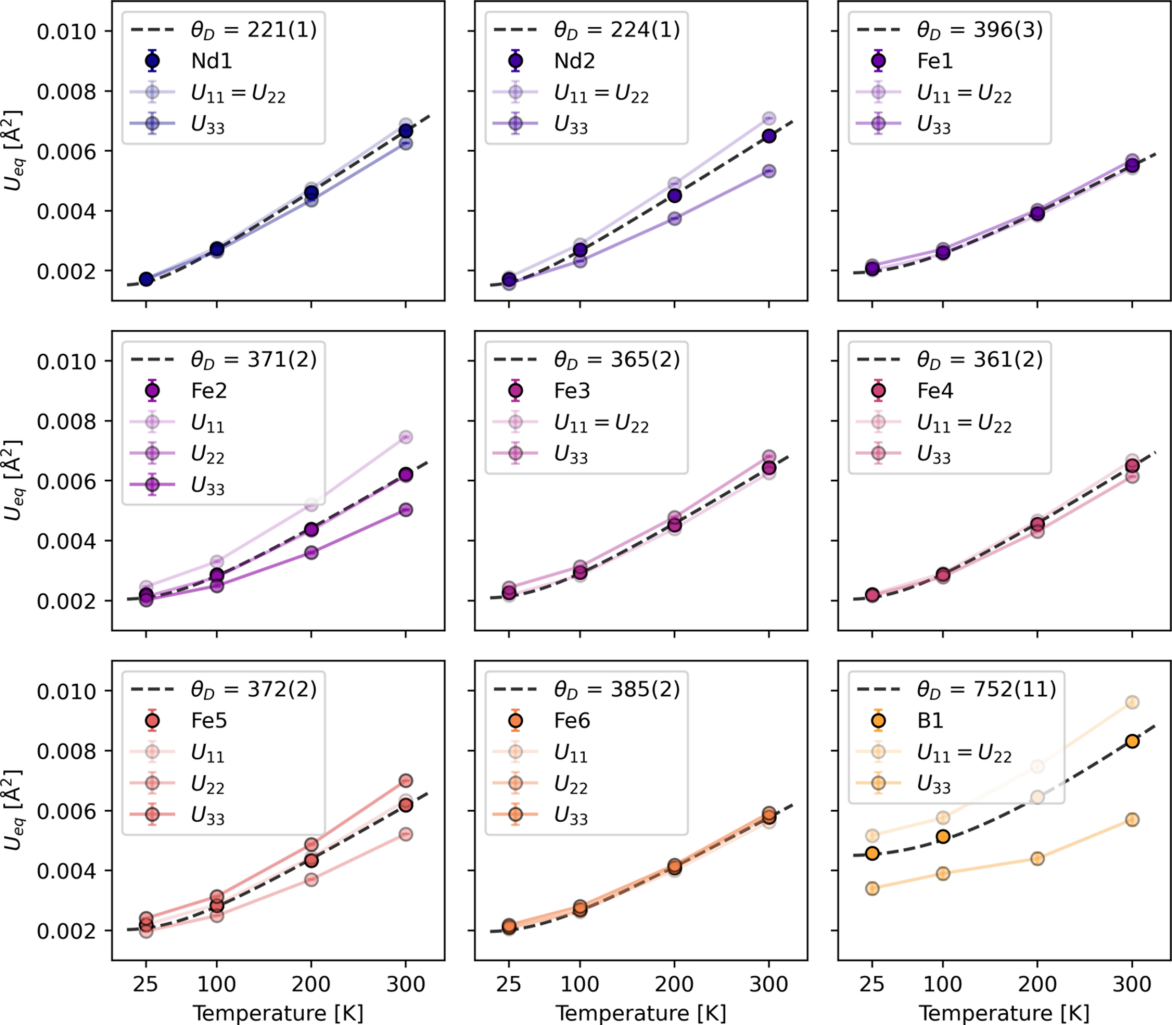
Vibrational parameters (*U_ij_*) for the nine atoms in the asymmetric unit. The full lines show *U*_eq_ while the shaded lines show vibrations along each of the principal axes of the ellipsoid. The dotted black line is the model fit to the 25–300 K *U*_eq_ data points. Some directions are restricted by the site symmetry. Debye temperatures in Kelvin are given for each atom.

The evolution of vibrational parameters (*U*_*ij*_) from 25 K to 300 K for each atom in the asymmetric unit can be seen in Fig. 4 ▸ along with calculated values for *U*_eq_. The Debye temperature (θ_D_) for each atom was calculated from the integral form of the Debye model fitted against *U*_eq_ data points from 25 K to 300 K (Willis & Pryor, 1975 ▸), as
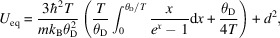
where ℏ is 1/2π times the Plank constant; *k*_B_ is the Boltzman’s constant; and *m* represents the atomic masses of 144.242 g mol^−1^, 55.845 g mol^−1^ and 10.811 g mol^−1^ for Nd, Fe and B, respectively. *d*^2^ is a term to describe the potential static disorder components of the ADP (Bentien *et al.*, 2005 ▸). Nd shows the lowest Debye temperature of ∼220 K. The average value for Fe is 375 K, while B has a much higher Debye temperature than the other atoms of 752 (11) K, as expected due to tighter bonding and a more rigid local framework surrounding B.

Fits to individual atom ADPs is not the intended use of the Debye model developed for a monoatomic cubic lattice, but still provides some insights into the thermal behaviour of each atom in the material (Willis & Pryor, 1975 ▸; Bentien *et al.*, 2005 ▸). For a polyatomic solid there is no straightforward way of calculating the material’s Debye temperature, but two attempts have been made here. Values of 345 (2) K and 383 (3) K are obtained from (1) the average of the mass-weighted *U*_eq_’s and (2) the average of the Debye temperatures. The values determined from the ADPs are somewhat lower than the Debye temperature reported from heat capacity measurements of 418 (12) K (Morishita *et al.*, 2020 ▸).

### 3.2. Extended structure/bonding features/frameworks

**Figure 5:**
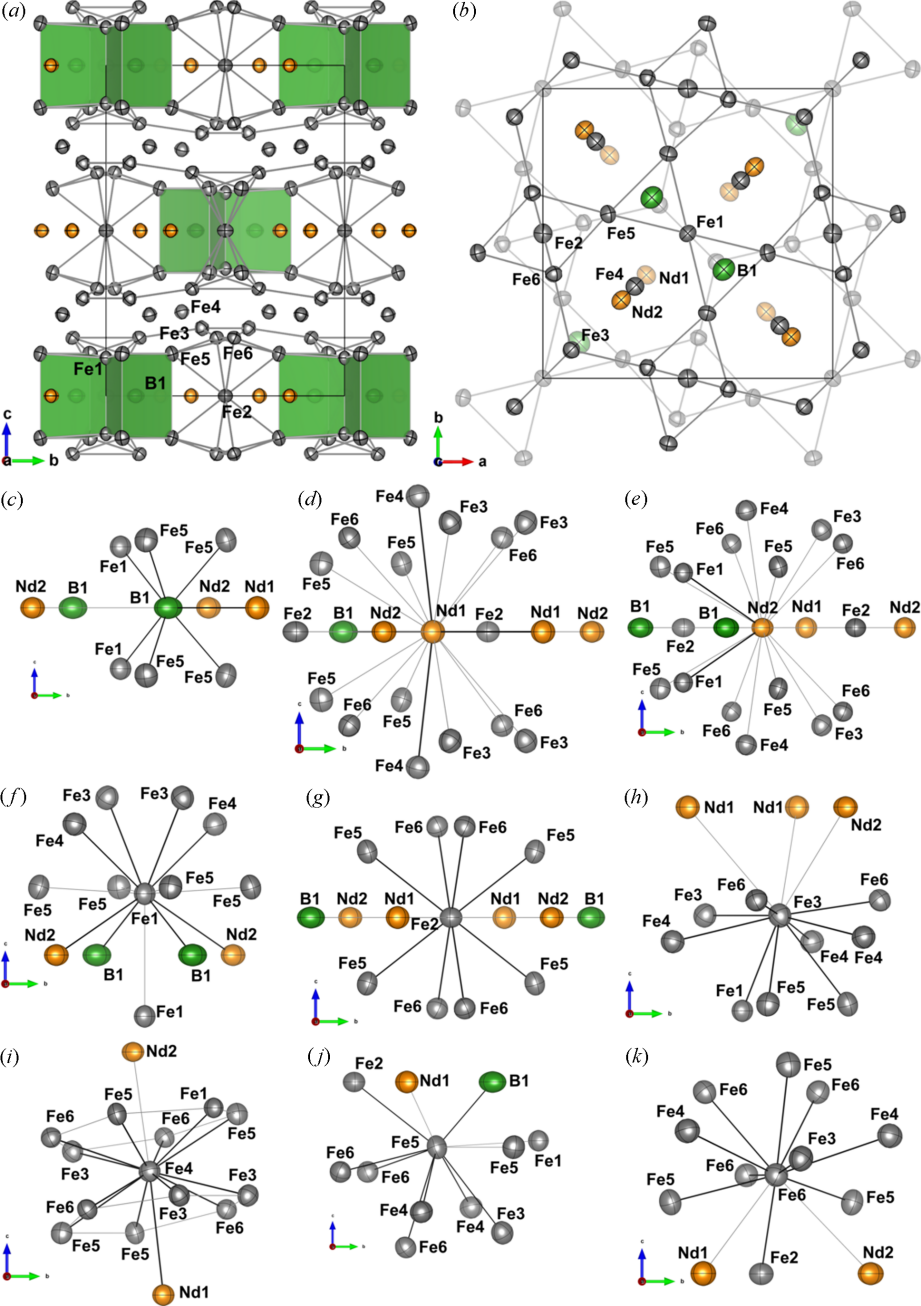
Unit cell along the (*a*) *a* and (*b*) *c* directions. Grey lines highlight the triangular-hexagonal Fe1/Fe3/Fe5/Fe6 layers with Fe4 in between and the Fe2 coordination. Green trigonal prisms show coordination of B. (*c*)–(*k*) Coordination environments of all symmetry-independent atoms visualized along the *a* direction. Thin grey lines show the first coordination sphere (within 3.8 Å), black lines show bonding contacts confirmed by the presence of BCPs at 25 K. The 300 K structure is used for visualization with thermal ellipsoids shown at a 99% probability level. Figures were drawn using *Vesta* (Momma & Izumi, 2008 ▸).

The unit cell, seen in Fig. 5 ▸, consists of alternating Fe and Nd/B layers. The description of the structure given here is inspired by the interpretation by others (Herbst *et al.*, 1985 ▸; Grin *et al.*, 2007 ▸). B1 forms distorted trigonal prisms with Fe1 and Fe5 in the vertices [green polyhedra in Fig. 5 ▸(*a*)]. Fe1/Fe3/Fe5/Fe6 form a triangular-hexagonal layer at *z* ≃ 1/3 (thickness in *z* < 0.1) with bond lengths of approximately 2.5 Å, shown in Fig. 5 ▸(*b*). Fe4 is placed in the *z* = 1/4 plane between two triangular-hexagonal layers in *z* ≃ 1/6 and *z* ≃ 1/3 with slightly longer bond lengths of 2.7 Å to each layer. The triangular-hexagonal layers have two types of hexagons (containing the atoms: Fe3/Fe5/Fe6 and Fe1/Fe3/Fe5/Fe6). Each Fe4 coordinates to two hexagons of different types in the layers above and below as shown in Figs. 5 ▸(*b*) and 5 ▸(*i*). The two triangular-hexagonal sandwich layers are related by a 90° rotation and a translation of 1/2 along the *ab* diagonal. Together each triangular-hexagonal layer forms a slightly distorted trigonal framework with Fe4. Fe2 is placed in the Nd/B layer symmetrically coordinating to 8 Fe atoms (two Fe5 and two Fe6 below and above the *z* plane) linking two trigonal Fe sandwich frameworks, as shown in Fig. 5 ▸(*a*). All interatomic vectors between Nd and Fe have a low difference of mean-square displacement amplitude (DMSDA) on the order of 10^−4^ Å^2^ while bonds with B, as expected, have one order of magnitude higher DMSDA. The bond lengths are in good agreement with Herbst *et al.* (1985 ▸) and Shoemaker *et al.* (1984 ▸).

### 3.3. Bonding analysis and electron density

Electron density analysis is a powerful tool to determine and categorize chemical bonds in crystals from diffraction data (Coppens, 1997 ▸). Chemical bonds and other topological features are well defined within the quantum theory of atoms in molecules (QTAIM) (Bader, 1994 ▸). Bond critical points (BCPs) and their properties can be found in Table 3 ▸. Only few have attempted modelling the electron density of lanthanide and actinide compounds (Eu, Gd, Tb, Dy, Th, U) and generally the *f*-block element is found surrounded by much less electron-rich ligands in a metal–organic complex (Iversen *et al.*, 1998 ▸; Iversen *et al.*, 1999 ▸; Gianopoulos *et al.*, 2017 ▸; Gianopoulos *et al.*, 2019 ▸; Zhurov, Zhurova & Pinkerton, 2011 ▸; Zhurov, Zhurova, Stash & Pinkerton, 2011 ▸; Gao *et al.*, 2020 ▸; Ananyev *et al.*, 2016 ▸; Puntus *et al.*, 2008 ▸). Exact determination of the coordination number of atoms in especially polyatomic solids is troublesome and depends on several assumptions and definitions. Here the presence of a BCP shared between two atoms is considered a point of interaction and will be noted as a bond. A more rigid discussion about the chemical implications of BCPs, bond paths and the presence of shared interatomic surfaces between atoms in the topological framework has recently been given elsewhere (Wagner *et al.*, 2025 ▸).

**Figure 6:**
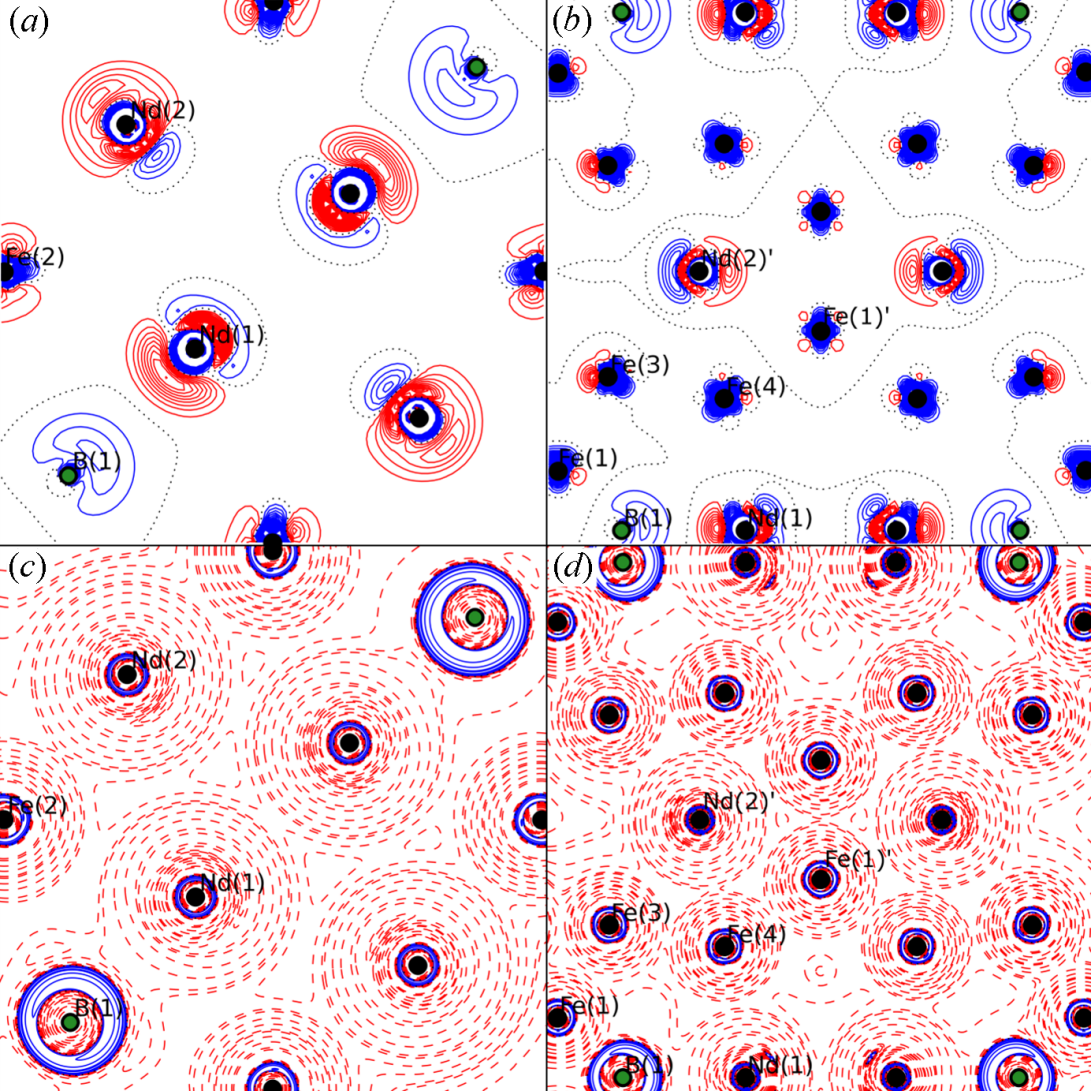
Deformation density (ρ_MM_ − ρ_IAM_) and the negative of the Laplacian (−∇^2^ρ_b_) in the (*a*) and (*c*) (001) plane, and (*b*) and (*d*) (110) plane. Contours are shown with 0.1 e Å^−3^ or exponential increments for the deformation density and Laplacian, respectively, positive is blue, negative is red.

Polarization of the outermost Nd core was observed as a necessity to refine a separate κ and dipole for the 4*d*^10^5*s*^2^5*p*^6^ shell. This may signify some involvement of these diffuse orbitals in the bonding, as previously observed for Th (Iversen *et al.*, 1998 ▸). One Nd—Nd bond with a distance of 3.57 Å is observed between two symmetry-related Nd1 atoms [Fig. 5 ▸(*d*)]. Regardless of the apparent high coordination number of the Nd atoms, only two Nd—Fe bonds are confirmed by BCPs, the Nd1—Fe4 and Nd2—Fe1 with bond lengths of 3.14 Å and 3.20 Å, respectively. Deformation density maps [Figs. 6 ▸(*a*) and 6 ▸(*b*)] are calculated by subtracting the IAM density from the MM density and show how the MM shifts the density from red regions to blue regions compared with the IAM. B1 clearly has a lone pair (blue contours in Fig. 6 ▸) towards Nd1 consistent with a BCP observed between the two atoms with a bond length of 2.90 Å [Figs. 5 ▸(*c*), 5 ▸(*d*) and 6 ▸(*a*)]. The B1—Fe1 and B1—Fe5 bonds have the shortest bond distances of approximately 2.1 Å with the highest electron density in the BCPs. Both Nd1 and Nd2 seem to deform along the *c* direction, relocating density from within the *z* = 0 plane to above and below. The effect is most pronounced for Nd1 in Fig. 6 ▸(*b*), where pronounced peaks (blue contours, not enclosing the atom centre) of density are seen shifted towards the centre of the cell, slightly above and below *z* = 0. The same holds true for most of the Fe atoms [Fig. 6 ▸(*b*)].

#### 3.3.1. Analysis of topological features

Herbst *et al.* (1985 ▸) hypothesized that the magnetic moment direction along the *c* axis (easy axis) originates from the anisotropic Fe sublattice. They identify that in general a given Fe atom is coordinated to more Fe neighbours above than below the *ab* plane in which the Fe atom is placed. The opposite arrangement (with more neighbours below than above) is also found within the unit cell, due to the space-group symmetry (a mirror plane in *z* = 0.5), but the argument with locally anisotropic structural features in the *c* direction still holds true.

Fe1 should have 11 contacts in total, 9 Fe: 1 below, 4 in plane and 4 above. The four interactions with Fe3 and Fe4 with distances of 2.52 Å and 2.79 Å, respectively, above the plane were confirmed by the presence of BCPs [Fig. 5 ▸(*f*)]. No BCPs were found between Fe1 and Fe5 in the plane, and little to no deformation is observed for either atom in the direction of the interaction. A ring critical point (3, +1) was found in (0, 0, 0) between Fe1 and the symmetry-related Fe1 below, which makes sense since it is a high-symmetry point enclosed by Fe1, B1 and Nd2, but no BCP was found to confirm the Fe1—Fe1 bond. Regardless, some asymmetry is still confirmed in the deformation density map [Fig. 6 ▸(*b*)] where the density on Fe1 in the (110) plane is seen to form some mushroom shape in the *c* direction. In general, the clearest electron deformation relative to the IAM is observed for the Nd and B atoms, Fig. 6 ▸.

Topological analysis of the coordination environment around Fe2 shows the expected BCPs to Fe5 (2.60 Å) and Fe6 (2.50 Å) symmetrically above and below the plane, in total eight bonds [Fig. 5 ▸(*g*)]. Fe3 coordinates to nine Fe atoms (Fe1, Fe3, Fe4, Fe5 and Fe6) in and below the plane with distances between 2.40 Å and 2.78 Å [Fig. 5 ▸(*h*)]. Fe4 coordinates to six Fe above and six Fe below [Fig. 5 ▸(*i*)]. All 12 Fe4—Fe interactions have distances between 2.64 Å and 2.79 Å with BCPs confirming the structural predictions by Herbst *et al.* (1985 ▸). Within each of the rings the expected bonds are found. According to Herbst *et al.* (1985 ▸) Fe5 should coordinate to 11 atoms in total, and 9 Fe: 1 below, 2 in plane and 6 above [Fig. 5 ▸(*j*)]. All out-of-plane interactions (2.52–2.76 Å) were confirmed by BCPs, only the in-plane interactions with Fe1 and Fe5 interactions are missing. This aligns with what was observed for Fe1 and confirms the local asymmetry in Fe coordination. All 10 expected Fe interactions (Fe2, Fe4, Fe5 and Fe6) with Fe6 are confirmed by BCPs (2.40–2.67 Å) expressing local anisotropy [Fig. 5 ▸(*k*)].

Herbst *et al.* (1985 ▸) and Shoemaker *et al.* (1984 ▸) differ in only two aspects: (1) Herbst *et al.* (1985 ▸) did not consider a bond between Nd1 and Nd2 while Shoemaker *et al.* (1984 ▸) did, and (2) Herbst *et al.* (1985 ▸) reported that the Fe1—Fe1 distance is shorter than the Fe1—Fe4 separation and that there is a bond. We agree with Herbst *et al.* (1985 ▸) that there is no bond between Nd1 and Nd2, but find an Nd1—Nd1 BCP. For the Fe1 interactions, we find that the Fe1—Fe4 distance [2.77275 (5) Å] is shorter than the Fe1—Fe1 distance [2.81971 (7) Å], in agreement with Shoemaker *et al.* (1984 ▸). This is further supported by a BCP between Fe1 and Fe4, where none is present between Fe1 and Fe1.

#### 3.3.2. Classification of bonds

Based on the dichotomous classification depending on the sign of ∇^2^ρ_b_, all interactions are closed-shell (∇^2^ρ_b_ > 0, |λ_1,2_|/λ_3_ ≪ 1, ρ_b_ ≃ 0.1–0.5 e Å^−3^) ionic or van der Waals (vdW) interactions (Gatti, 2005 ▸). Interactions between B and Fe have higher ρ_b_ ≃ 0.5 e Å^−3^ and ∇^2^ρ_b_ ≃ 3.5 e Å^−5^, approaching values for metal–ligand interactions in transition metal complexes (Grønbech *et al.*, 2023 ▸, Macchi *et al.*, 1998 ▸), while the other interactions have lower values: Nd1—Nd1 0.095 e Å^−3^ (0.468 e Å^−5^), Nd—Fe ≃ 0.15 e Å^−3^ (0.8–1.0 e Å^−5^) and Fe—Fe 0.23–0.34 e Å^−3^ (1.0–2.3 e Å^−5^). Fe—Fe bonds have values consistent with other reports of transition metal–transition metal bonds in metal complexes (Macchi *et al.*, 1998 ▸). The bond distances are correspondingly short for B—Fe (2.10 Å), slightly longer for Fe—Fe (≃2.5–2.8 Å) and longest for interactions with Nd (>3.0 Å).

Kinetic (*G*_b_), potential (*V*_b_) and total (*H*_b_) energy densities can be estimated from the density (ρ_b_) and Laplacian (∇^2^ρ_b_) values at the BCP according to the Abramov (1997 ▸) approximation,
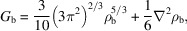
and the local virial theorem (Bader, 1994 ▸),
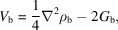
while the total energy is simply a sum of the kinetic and potential energies. Using the energy densities at the BCPs, the bonding analysis can be expanded to the classification by Espinosa *et al.* (2002 ▸) based on the |*V*_b_|/*G*_b_ ratio. In this classification, all interactions in Nd_2_Fe_14_B are placed in the transit region between shared-shell and closed-shell interactions with 1 < |*V*_b_ |/*G*_b_ < 2, *H*_b_ < 0 and ∇^2^ρ_b_ > 0. All observed interactions have |*V*_b_ |/*G*_b_ ≃ 1.1–1.5 and *H*_b_ < 0.1 e Å^−3^ (except Fe—B interactions with *H*_b_ ≃ 0.15 e Å^−3^), well in line with the nature of other metal–metal bonds in crystals. The bond degree (*H*_b_/ρ_b_) approaches zero at the boundary with the closed-shell region, suggesting a more closed-shell nature of the Nd bonds (|*H*_b_/ρ_b_| ≃ 0.05) than the Fe—Fe (|*H*_b_/ρ_b_| ≃ 0.1–0.25) and Fe—B with |*H*_b_/ρ_b_| > 0.3, suggesting a bond degree of 1/3 of a single bond. Classification of bonds based on the properties in the BCPs aligns well with the observed Laplacian maps in Figs. 6 ▸(*c*) and 6 ▸(*d*).

#### 3.3.3. Atomic charge and *d*-orbital populations

Charge transfer between atoms is allowed in the MM and integration of the atomic basin gives the Bader charge and other associated atomic properties. It is found that both Nd atoms are positive, +0.91 and +1.05 for Nd1 and Nd2, respectively, and B is negative, −1.7. Fe1, Fe2, Fe3, Fe5 and Fe6 are predominantly neutral, approximately ±0.1, with Fe1 almost exactly neutral (+0.04), while Fe4 is negative −0.44. Empirically, lanthanides have the +III oxidation state, while B is preferably negatively charged, although maximally −III. Fe is typically positively charged with a range of possible oxidation states. Obeying the preferred formal charges of Nd and B would leave the 14 Fe atoms with a formal charge of −0.2. Other rationalizations could be (1) Fe atoms of different charges; and/or (2) neutral Fe atoms accompanied by a reduced charge of Nd, as observed in this study. The calculated Bader charges show larger absolute values for Nd and B compared with Fe, meaning that the primary charge transfer in this compound comes from donation of electrons from Nd to B. Debye temperatures, *d*-orbital populations, Bader charges, atomic dipole moments and atomic volumes for the Fe atoms can be seen in Table 4 ▸.

The *d*-orbital populations are approximately six electrons for all Fe atoms and are quite spherically distributed. A fully spherical *d*-orbital splitting would arise from a 20% occupancy of each *d* orbital. The asphericity, α, is calculated as the mean square error: 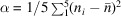, a sum over the five *d* orbitals where *n_i_* is the percentage occupancy of the *i*th orbital and 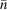 is the mean occupancy assuming spherical distribution (20%). For comparison, the asphericity with six *d* electrons in high-spin configuration of some common coordination environments is approximately an order of magnitude higher than what we observe here: perfect linear, α = 16.7; perfect octahedral, α = 7.4; and experimental octahedral, α = 4.9 [calculated from the experimental *d*-orbital occupancies of FeSb_2_ (Grønbech *et al.*, 2020 ▸)]. Our results vary between 0.46 and 1.50, meaning that almost no *d*-orbital aphericity is observed.

The theoretical maximal spin-only magnetic moment, μ_S_, can be calculated as 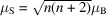, where *n* is the number of unpaired electrons. Assuming a high-spin configuration, the maximal μ_S_ would be reached for five unpaired electrons; for the *d*^6^ configuration, approximately observed for the Fe ions here, the expected maximal spin-only moment would be 4.9 μ_B_. Table 4 ▸ shows that Fe4 has the highest *d*-orbital population of 6.27 electrons, which would mean that Fe4 has the lowest magnetic moment, in contradiction to neutron experiments (Herbst *et al.*, 1985 ▸) and theoretical calculations (Gu & Ching, 1987 ▸; Toga *et al.*, 2016 ▸). From the generally much lower experimentally observed magnetic moment (Table 4 ▸), compared with the theoretical spin-only moment, we can conclude that either the simple spin-only model or the orbital-splitting model is too crude an approximation to reflect the magnetism in Nd_2_Fe_14_B.

The lowest atomic volumes are found for Fe1 and Fe5, both with relatively short bonds to B. Atomic vibrations are very similar for all Fe atoms at 25 K, with the lowest Debye temperature being that of Fe4 [361 (2) K] and highest that of Fe1 [396 (3) K]. Fe4 has a remarkably low electric dipole moment of 0.28 × 10^−30^ C m, while Fe3 has a moment as high as 4.4 × 10^−30^ C m, serving as evidence of the very symmetric or asymmetric coordination environment, respectively. No single parameter seems to be a strong descriptor for the varying magnetic moments between the Fe atoms.

### 3.4. Structure and magnetism

Nd_2_Fe_14_B is a metal with resistivity (ρ = 130–150 µΩ cm at room temperature) only slightly higher than many *d*-block metals (Bovda *et al.*, 2006 ▸; Stankiewicz & Bartolomé, 1999 ▸). The magnetism in Nd_2_Fe_14_B can be described as a 4*f*–3*d* exchange interaction between the lower lying 4*f* electrons of Nd and the 3*d* electrons of Fe close to the Fermi level (Min *et al.*, 1993 ▸). It is generally accepted that the magnetism of rare-earth intermetallic compounds is very complex and that the resulting magnetic ordering arises from contributions from both types of electrons. The magnetic ordering can be viewed as a 4*f* stabilization of the 3*d* free electron sea, meaning that the Fe framework forms a metallic magnetic pathway with contributions from the Nd electrons residing in the 4*f* orbitals.

It is well established that the crystallographic *c* direction is the easy axis of magnetization, and that the strong magnetic moment originates mainly from the Fe sublattice (Herbst, 1991 ▸; Hirosawa *et al.*, 1986 ▸; Sagawa *et al.*, 1985 ▸; Givord *et al.*, 1984 ▸; Sinnema *et al.*, 1984 ▸). Fe4 possess the strongest magnetic moment (2.7–3.5 µB) of all Fe atoms with the rest being only slightly smaller on average, ∼2.2 µB (Tharp *et al.*, 1987 ▸; Toga *et al.*, 2016 ▸). The local anisotropic coordination along the *c* direction for all Fe atoms is assumed to improve the magnetic interactions (Herbst *et al.*, 1985 ▸). The strong preference for magnetic stability along the *c* direction was reported to arise from Nd, with a magnetic moment (3.27 µB) close to that of a free Nd^3+^ ion (Sagawa *et al.*, 1985 ▸; Givord *et al.*, 1984 ▸), specifically Nd1 was found to stabilize and dictate the magnetic easy axis (Haskel *et al.*, 2005 ▸). Several reports note that Nd and Fe4 must have a favourable strong interaction due to the much higher magnetic moment of Fe4 in Nd_2_Fe_14_B than in Y_2_Fe_14_B (Gu & Ching, 1987 ▸; Wolfers *et al.*, 2001 ▸). This fits well with the BCP observed here between Nd1 and Fe4. Fe atoms surrounded by B should have a lower 3*d* magnetic moment due to an ineffective 2*p*–3*d* charge transfer/hybridization, which explains the low magnetic moment of Fe1 (Givord *et al.*, 1985 ▸).

**Figure 7:**
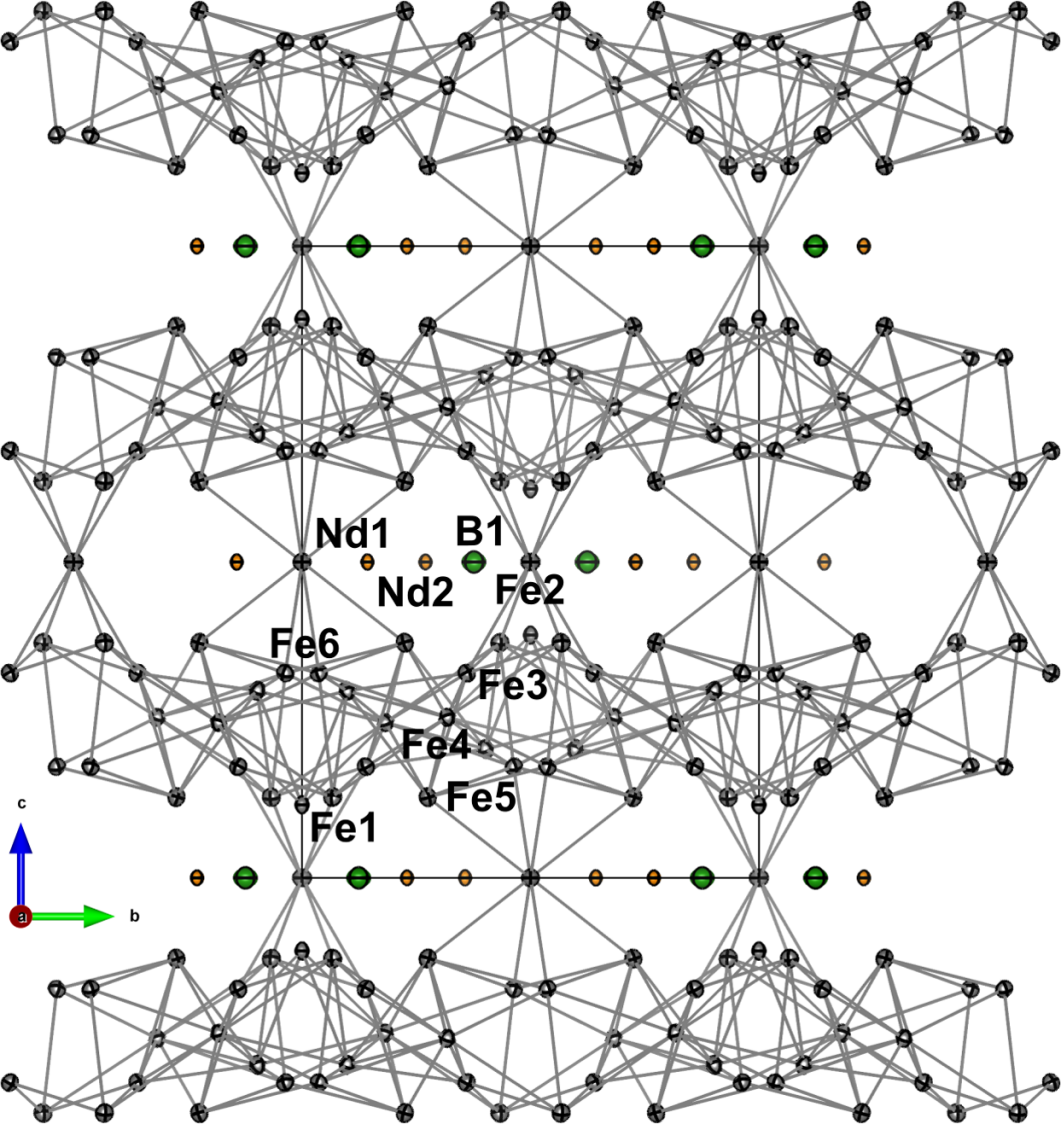
Molecular graph showing only the Fe–Fe interactions with grey lines indicating bonds with BCPs. The 25 K structure is used for visualization with thermal ellipsoids shown at a 99% probability level.

The molecular graph of Nd_2_Fe_14_B in Fig. 7 ▸ (showing only Fe—Fe bonds with BCPs) reveals a comprehensive framework of interactions. Each Nd contributes with only one bond to the Fe framework: Nd2—Fe1, where the interaction lies almost in the *ab* plane, and Nd1—Fe4, which lies along the *c* direction. Except for the interactions with Nd, Fe2 stands out as the crucial link to facilitate a pathway along the *c* direction. Without this link, the interaction would be solely two-dimensional within the Fe sandwich layers. None of the Fe atoms in the Fe sandwich layers (Fe1, Fe3, Fe4, Fe5 or Fe6) serve as crucial a role as Fe2. Removing either one of the Fe atoms will not reduce the dimensionality of the framework further, and while Fe4 still stands out as the atom with most interactions and a negative charge, even the link between the triangular-hexagonal layers is maintained without Fe4. The bonds determined by topological analysis of the electron density agree with the relative strength of magnetic interactions between different crystallographic sites as reported by Bouaziz *et al.* (2023 ▸). Relatively few electron density studies have been performed on metallic solids (Iversen *et al.*, 1995 ▸; Bader, 1994 ▸). These usually crystallize in highly symmetric space groups with few degrees of freedom, which also restricts the topological fingerprint. Nd_2_Fe_14_B crystallizes in the tetragonal crystal system. The unusually high number of independent atoms in the asymmetric unit (two Nd, six Fe and B) provides abundant degrees of freedom to describe the comprehensive topology of the metal.

## 4. Conclusions

Multi-temperature synchrotron SCXRD data have been measured on the super-strong permanent magnet Nd_2_Fe_14_B. Despite a very low X-ray electron density suitability factor, it was possible to obtain a multipole electron density model at 25 K. A linear, though minute, expansion of the unit cell is observed in the *c* direction [6.6 (7) × 10^−6^ K^−1^], while the *ab* plane remains unchanged in the investigated temperature range from 25 K to 300 K. In general, very few Nd_2_Fe_14_B structures are reported with vibrational parameters, and those attempting a refinement significantly overestimate the vibrations of B. Here we report vibrational parameters of *B*_eq_ ≃ 0.5 Å^2^ for Nd and Fe, and *B*_eq_ = 0.658 (7) for B at 300 K. The material’s Debye temperature was estimated from vibrational parameters to be 345–383 K, which is lower than the 418 (12) K reported from heat capacity measurements.

A multipole electron density was derived in line with the Hansen–Coppens multipolar formalism with *l*_max_ = 4. Satisfactory modelling of the Nd atoms required a dipole deformation of the core. Deformation densities and QTAIM analysis of the topological features of the electron density show local anisotropy in the bonding environment of the Fe atoms, confirming bonding information suggested by Herbst *et al.* (1985 ▸) based on atom proximity. By Bader topological analysis it was found that Nd atoms are positive (∼+1), B and Fe4 are negative (−1.7 and −0.44, respectively) and the remaining Fe atoms are neutral (±0.1). The *d* orbitals of Fe are close to evenly populated. The molecular graph showing a comprehensive framework of Fe interactions, with very little influence from the Nd atoms, serves as a visual chemical descriptor of the 4*f*–3*d* exchange magnetic ordering interaction in Nd_2_Fe_14_B and highlights the crucial role of Fe2 on the 3D properties of the framework.

## Supplementary Material

Crystal structure: contains datablock(s) nd2fe14b_100k, nd2fe14b_200k, nd2fe14b_300k, XD. DOI: 10.1107/S2052252525007602/fc5084sup1.cif

Supporting information. DOI: 10.1107/S2052252525007602/fc5084sup2.pdf

CCDC references: 2485375, 2485376, 2485377, 2485378

## Figures and Tables

**Table 1:** Crystallographic information for Nd_2_Fe_14_B in the tetragonal space group *P*4_2_/*mnm* with *Z* = 4 Values for the 25 K dataset are given for the MM from *XD2016* (Volkov *et al.*, 2016 ▸), while the others are for the IAM in *Olex2* (Dolomanov *et al.*, 2009 ▸). The definition of parameters varies slightly and should therefore not be compared between programs.

	Temperature (K)			
	25	100	200	300
*a*, *c* (Å)	8.8128 (2), 12.1904(3)	8.8135 (2), 12.1933 (3)	8.81530 (14),† 12.2028 (2)	8.8143 (2),† 12.2117 (2)
*V* (Å^3^)	946.77 (5)	947.15 (5)	948.27 (3)	948.75 (5)
Measured, independent reflections	371711, 7099	143660, 4151	143509, 4167	142676, 4168
*R*_int_, multiplicity, completeness (%)	0.046, 42.4, 99.9	0.042, 34.5, 100.0	0.047, 34.4, 100.0	0.037, 34.2, 100.0
*R*(*F*^2^), *wR*(*F*^2^), GooF	0.014, 0.030, 1.00	0.009, 0.021, 1.12	0.009, 0.022, 1.07	0.008, 0.020, 1.13
Δρ_max_, Δρ_min_ (e Å^−3^)	1.25, −2.13	0.88, −2.50	0.75, −2.31	0.62, −1.75

† The minute reduction observed for the *a* lattice parameter from 200 K to 300 K is attributed to the relatively lower experimental precision in unit-cell determination by SCXRD.

**Table 2:** Atomic positions and *B*_eq_ at 300 K Coordinates are reported for positions equivalent with Table 1 in Shoemaker *et al.* (1984 ▸) and *B*_eq_ is derived from *U_ij_* as *B*_eq_ = 8π^2^*U*_eq_, where *U*_eq_ = (*U*_11_ + *U*_22_ + *U*_33_)/3 for an easier comparison. Unit-cell transformation: rotation: (0, 1, 0; −1, 0, 0; 0, 0, 1) and translation: (0, 0, 1/2).

	*x*, *y*, *z*	*B*_eq_ (Å^2^)
Nd1	0.14309 (2), 0.14309 (2), 0.00000	0.5266 (8)
Nd2	0.72978 (2), 0.27022 (2), 0.00000	0.5140 (8)
Fe1	0.00000, 0.00000, 0.38499 (2)	0.4358 (8)
Fe2	0.00000, 0.50000, 0.000000	0.4911 (8)
Fe3	0.40196 (2), 0.40196 (2), 0.29525 (2)	0.5085 (8)
Fe4	0.18254 (2), 0.18254 (2), 0.25424 (2)	0.5132 (8)
Fe5	0.72459 (2), 0.06700 (2), 0.37236 (2)	0.4887 (8)
Fe6	0.46308 (2), 0.13995 (2), 0.32395 (2)	0.4564 (8)
B1	0.37563 (7), 0.37563 (7), 0.00000	0.658 (7)

**Table 3:** Bond critical points (BCPs) and their properties as determined by topological analysis of the electron density Derived properties are calculated from the density, ρ, and Laplacian, ∇^2^ρ, using the Abramov approximation and the virial theorem for the kinetic energy, *G*, and potential energy, *V*, respectively (Gatti, 2005 ▸). The unit H is Hartree. Uncertainties on ρ and ∇^2^ρ are ±0.001 as estimated by *XD2016*, while uncertainties on the energies found by propagation of error are on the second decimal. These values are much lower than typical systematic errors in the X-ray electron density analysis (Shi *et al.*, 2019 ▸).

		ρ_b_ (e Å^−3^)	∇^2^ρ_b_ (e Å^−5^)	*R_ij_* (Å)	ɛ	*G*_b_ (H Å^−3^)	*V*_b_ (H Å^−3^)	*H*_b_ (H Å^−3^)	*H*_b_/ρ_b_	|*V*_b_|/*G*_b_
Nd1	Nd1^i^	0.095	0.468	3.566	0.84	0.04	−0.04	0.00	−0.05	1.13
	B1	0.221	1.613	2.904	0.22	0.14	−0.17	−0.03	−0.12	1.19
	Fe4	0.168	1.016	3.137	0.09	0.09	−0.11	−0.02	−0.10	1.20
Nd2	Fe1^ii^	0.132	0.799	3.197	1.19	0.06	−0.07	−0.01	−0.07	1.14
B1	Fe1	0.464	3.401	2.095	0.06	0.38	−0.53	−0.14	−0.31	1.38
	Fe5^iii^	0.528	3.588	2.110	0.04	0.44	−0.64	−0.19	−0.37	1.44
Fe1	Fe4^iii^	0.225	1.085	2.787	0.1	0.12	−0.16	−0.04	−0.18	1.35
	Fe3	0.282	1.928	2.516	0.18	0.19	−0.24	−0.05	−0.19	1.28
Fe2	Fe5^iv^	0.229	1.530	2.597	0.31	0.14	−0.17	−0.03	−0.15	1.24
	Fe6	0.260	1.965	2.498	0.02	0.18	−0.22	−0.04	−0.15	1.22
Fe3	Fe3^v^	0.337	1.911	2.444	0.1	0.22	−0.31	−0.09	−0.26	1.39
	Fe5^vi^	0.280	1.483	2.593	0.19	0.17	−0.23	−0.06	−0.22	1.37
	Fe4	0.257	0.955	2.784	0.57	0.13	−0.19	−0.06	−0.24	1.48
	Fe6^vii^	0.344	2.338	2.399	0.04	0.24	−0.33	−0.08	−0.24	1.33
Fe4	Fe5	0.246	1.038	2.759	0.08	0.21	−0.22	−0.01	−0.05	1.06
	Fe6	0.277	1.211	2.666	0.32	0.22	−0.25	−0.03	−0.12	1.15
Fe5	Fe6^viii^	0.293	1.777	2.522	0.13	0.22	−0.27	−0.05	−0.15	1.20
Fe6	Fe6^ix^	0.289	1.653	2.549	0.04	0.22	−0.26	−0.04	−0.15	1.19

Symmetry codes: (i) 1 − *x*, 1 − *y*, −*z*; (ii) 1 + *x*, *y*, *z*; (iii) 1/2 − *y*, *x* − 1/2, 1/2 − *z*; (iv) 1/2 − *x*, 1/2 + *y*, 1/2 − *z*; (v) −*x*, −*y*, *z*; (vi) *x* − 1/2, 1/2 − *y*, 1/2 − *z*; (vii) *y* − 1/2, 1/2 − *x*, 1/2 − *z*; (viii) *y*, *x*, *z*; (ix) 1/2 − *y*, 1/2 + *x*, 1/2 − *z*.

**Table 4:** *d*-orbital populations for the Fe atoms derived from the multipole populations Percentage occupancy is given in parenthesis. The local coordinate system for each Fe atom is placed with the *z* axis parallel to *c* and the *y* axis parallel to (110), except for the Fe4 coordination environment, which is tilted by ∼16° from the *c* axis to match the linear axis between the centre of the two Fe hexagons above and below. Integrated properties of the atomic basin are reported without uncertainties, but the integrated absolute Lagrangian, *L* (which should ideally be below 10^−4^ a.u.) is on the order of 10^−3^–10^−5^ a.u. (Volkov *et al.*, 2016 ▸).

	Fe1	Fe2	Fe3	Fe4	Fe5	Fe6
*z* ^2^	1.25 (21.0)	1.22 (20.4)	1.17 (19.8)	1.26 (20.1)	1.18 (20.4)	1.18 (20.1)
*xz*	1.15 (19.3)	1.24 (20.8)	1.26 (21.4)	1.37 (21.8)	1.18 (20.4)	1.21 (20.7)
*yz*	1.23 (20.6)	1.22 (20.4)	1.26 (21.4)	1.27 (20.3)	1.23 (21.2)	1.23 (21.0)
*x*^2^ − *y*^2^	1.16 (19.4)	1.11 (18.6)	1.09 (18.4)	1.16 (18.5)	1.06 (18.3)	1.12 (19.1)
*xy*	1.18 (19.7)	1.18 (19.8)	1.12 (19.0)	1.21 (19.3)	1.14 (19.7)	1.12 (19.1)
Total *d*_pop_	5.97	5.97	5.90	6.27	5.79	5.86
Asphericity, α	0.46	0.59	1.50	1.22	0.95	0.62
Bader (e)	+0.04	−0.09	−0.09	−0.44	+0.17	+0.11
Lagrangian, *L* (10^−3^ a.u.)	3.1	7.9	0.035	3.2	0.64	2.3
Dipole moment (10^−30^ C m)	1.45	1.02	4.40	0.28	1.34	1.56
Atomic volume (Å^3^)	11.81	14.95	14.71	14.25	12.45	13.04
*B*_eq_ at 25 K (Å^2^)	0.1634 (8)	0.1745 (8)	0.1777 (8)	0.1729 (8)	0.1729 (8)	0.1666 (8)
Debye temperature (K)	396 (3)	371 (2)	365 (2)	361 (2)	372 (2)	385 (2)
Coordination number	4 Fe, 2 B, 2 Nd	8 Fe	9 Fe	12 Fe, 1 Nd	7 Fe, 1 B	10 Fe
μ at 77 K†	1.1	2.2	2.7	3.5	2.4	2.4

† Reported by Herbst *et al.* (1985 ▸).
