# Supplementary material for: X-ray electron density analysis of chemical bonding in permanent magnet Nd2Fe14B
Source: IUCrJ. 2025 Sep 29;12(Pt 6):658–69. doi: 10.1107/S2052252525007602 (PMC12573920; doi:10.1107/S2052252525007602)
Supplement: Supplementary file 2 [file m-12-00658-sup2.pdf]

IUCrJ

Volume 12 (2025)

Supporting information for article:

X-ray electron density analysis of chemical bonding in permanent magnet Nd2Fe14B

Emilie Skytte Vosegaard, Jacob Svane and Bo Brummerstedt Iversen

S1. Core deformations

Three different models for core deformations were tested 1) no deformations (spherical core), 2) the outermost core orbitals with n=5: $5s^2 5p^6$ deformed by a dipole or 3) the model presented in the manuscript with $4d^{10} 5s^2 5p^6$ deformed by a dipole. Adding additional poles with l>1 gave no significant population terms and no improvement on the quality parameters/residuals, so only the dipole-deformed models will be discussed here. A comparison of quality parameters, min/max residuals and residual density maps in three different cases can be seen in Table S1. As can be seen from the quality parameters given in the table, the model significantly improved by including deformations for the $4d^{10} 5s^2 5p^6$ shell.

Table S1 Quality parameters for three models of core deformations: 1) no deformations (spherical) or including 2) $5s^2 5p^6$ or 3) $4d^{10} 5s^2 5p^6$.

Core model	**Spherical**	$\mathbf{5s^2 5p^6}$	$\mathbf{4d^{10} 5s^2 5p^6}$
R(F)[%]	1.01	1.01	0.97
R(F^2)[%]	1.50	1.52	1.44
GOF	1.0312	1.0315	0.9951
$\Delta\rho_{min}/\Delta\rho_{max}$ [e Å^{-3}]	-2.98/2.46	-2.83/2.30	-2.13/1.25

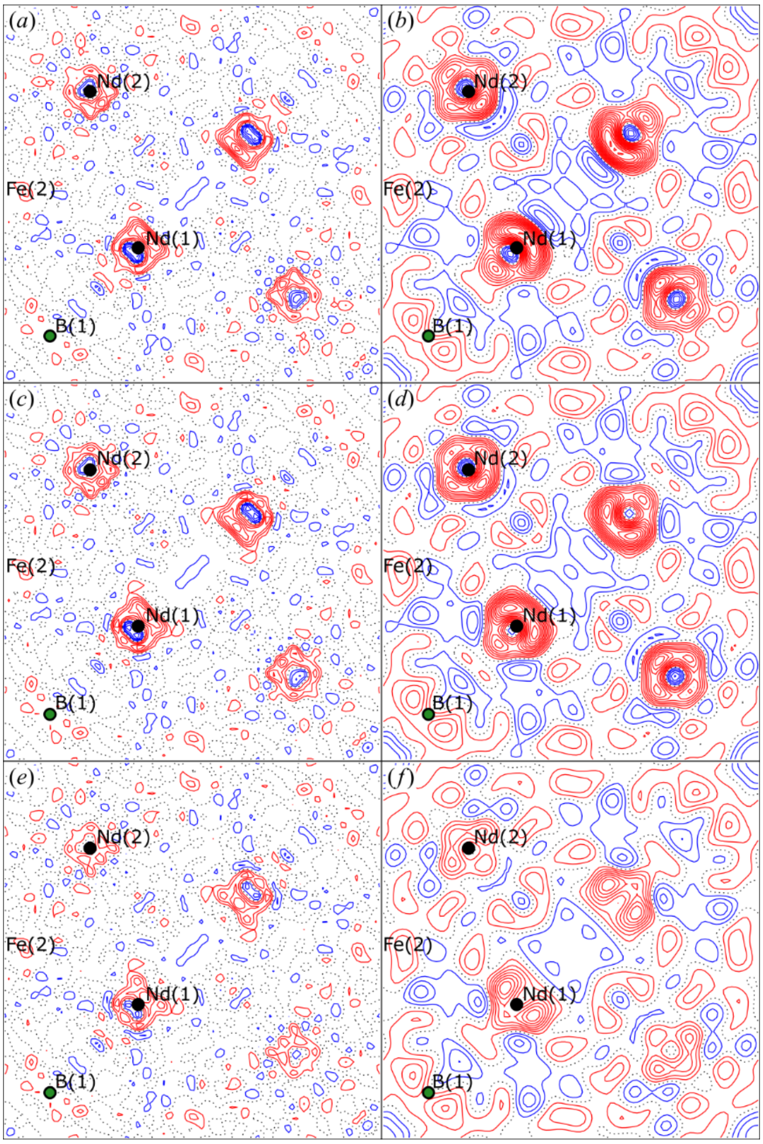

Figure S1 Residual density maps in the (001)- plane for the a-b) spherical model or including a dipole for the outermost core c-d) $5s^2 5p^6$ or e-f) $4d^{10} 5s^2 5p^6$ as indicated in Table S1. Residuals are shown to the full resolution (1.5 Å^{-1}) (left column) or a truncated resolution (0.7 Å^{-1}) to emphasize valence deformations (right column). Positive (blue), negative (red) and zero (black) contours are shown at steps of 0.5 e Å^{-3} for the full resolution plots or 0.1 e Å^{-3} level in the truncated case.

S2. Modelling f-electrons using an l_{max}=6 model

Jana2020 was used to construct two models for comparison with the XD2016 model, using either 4 or 6 as l_{max}, to determine the necessary highest poles. By including the same reflections and parameters in the model, equal models should be obtained in XD2016 and Jana2020 for the l_{max}=4 case. Any discrepancies between the two must be attributed differences in the programs. The Jana (l_{max}=4) model further serves as a reference for the Jana (l_{max}=6) model, to detect changes and improvements by increasing the number of poles. Due to technical differences in the two programs the models differ slightly in refined parameters e.g. dipole core deformations on Nd atoms and manual adjustments of the scale are not performed in Jana2020, which is

expected to lower the min and max residuals significantly (approx. $\Delta\rho_{min}$= -4.5 ⟶-2.4 e Å^{-3} and $\Delta\rho_{max}$=1.9→1.1 e Å^{-3} in the XD2016 model), but has no impact on the other refined parameters. Refinement parameters can be seen in Table S2.

The two l_{max}=4 models are very similar, while the Jana (l_{max}=4) and Jana (l_{max}=6) models can be considered essentially equal. Slight deviations are found for the Nd and Fe κ parameters. The monopole valences, P_{val}, are generally in good agreement between XD2016 and Jana2020. Using the monopole populations as an initial estimate of the atomic charge, the models seem to predict the same chemical trends. The Nd1, Nd2, Fe1, Fe2, Fe5 and Fe6 monopole populations are equal within the estimated uncertainties. Discrepancies are seen for the negatively charged B (and to some extent also Fe4), which are more negative in the Jana2020 models than the XD2016 model, while the positively charged Fe1, Fe2 and Fe3 are slightly more positive. These predictions are based on the assumption that the 4*s* population is 2 for all Fe atoms in the Jana2020 models. This assumption is significantly flawed, which can be seen for the refined 4*s*, P_{00}, population in the XD2016, varying between 1.1(7) and 2.8(4). Overall the models are in good agreement.

Figure S2 shows the residual density maps in the (001) and (1-10) planes of the unit cell. The residuals are remarkably similar across the models, and deviates only slightly on the Nd core sites. Based on these analysis increasing l_{max} to 6 gives no improvement over the presented l_{max}=4 model. This indicates that the residuals seen on Nd are unmodelled core deformations with negligible impact on the charge and bonding environment. It has furthermore been shown that the XD and Jana l_{max}=4 models have no significant discrepancies, as evidence of a robust model describing the experimental density well.

Table S2 Refinement parameters for the three models. *The Fe 4*s* populations were assumed to be 2 in the Jana2020 models.

Instrument	XD	Jana (l_{max}=4)	Jana (l_{max}=6)
R(F)[%]	0.97	1.04	1.04
R(F^2)[%]	1.44	3.42	3.41
GOF	0.9951	0.9048	0.90
$\Delta\rho_{min}/\Delta\rho_{max}$ [e Å^{-3}]	-2.13/1.25	-6.60/1.70	-6.61/1.71
$N_{unique}(F^2 > 3\sigma)$	6921	6921	6921
N_{obs}/N_{par}	55.4	55.4	49.8
Convergence crit.	0.00001	0.0005	0.0005
Scale	0.1615	1.620(4)	1.620(4)
κ(Nd)	1.049	1.160(12)	1.160(12)
κ(Fe)	1.046	0.994(3)	0.993(3)
κ(B)	0.990	0.94(4)	0.94(4)
P_{val}(Nd1)	5.80(12)	5.87(13)	5.86(13)
P_{val}(Nd2)	5.67(12)	5.61(12)	5.59(12)
P_{val}(Fe1)	5.97(8)	5.89(10)	5.85(10)
P_{val}(Fe2)	5.97(7)	5.78(12)	5.78(12)
P_{val}(Fe3)	5.88(5)	5.62(8)	5.62(8)

P_{val}(Fe4)	6.25(5)	6.52(8)	6.52(8)
P_{val}(Fe5)	5.78(5)	5.81(14)	5.81(15)
P_{val}(Fe6)	5.84(4)	5.81(6)	5.82(6)
P_{val}(B1)	4.2(3)	5.1(4)	5.1(4)
P_{00}(Fe1)	1.2(5)	2*	2*
P_{00}(Fe2)	1.1(7)	2*	2*
P_{00}(Fe3)	2.3(4)	2*	2*
P_{00}(Fe4)	2.8(4)	2*	2*
P_{00}(Fe5)	1.8(2)	2*	2*
P_{00}(Fe6)	2.0 (3)	2*	2*

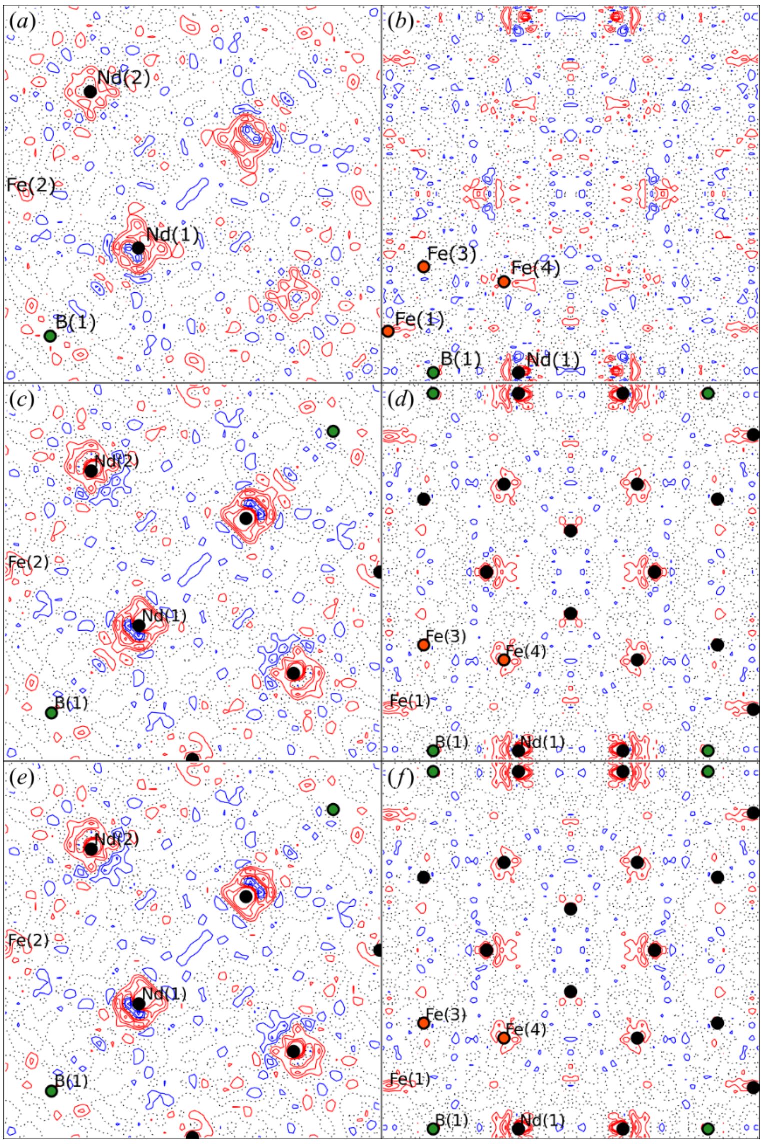

Figure S2 Residual density plots in the (001)- and (1-10)- planes (left and right columns respectively) for the full resolution 1.5 $Å^{-1}$ shown at 0.5 e $Å^{-3}$ intervals for the (a-b) XD model, (c-d) Jana l_{max}=4 model and (e-f) Jana l_{max}=6 model. Blue is positive, red is negative, black is zero.
